# Effects of an External Magnetic Field on the Interband and Intraband Optical Properties of an Asymmetric Biconvex Lens-Shaped Quantum Dot

**DOI:** 10.3390/nano12010060

**Published:** 2021-12-27

**Authors:** Mher A. Mkrtchyan, David B. Hayrapetyan, Eduard M. Kazaryan, Hayk A. Sarkisyan, Maxim Ya. Vinnichenko, Vadim A. Shalygin, Dmitry A. Firsov, Lyudvig S. Petrosyan

**Affiliations:** 1 General Physics and Quantum Nanostructures, Russian-Armenian University, 123 Hovsep Emin Str., Yerevan 0051, Armenia; mher.mkrtchyan@rau.am (M.A.M.); david.hayrapetyan@rau.am (D.B.H.); eduard.ghazaryan@rau.am (E.M.K.); 2 Institute of Electronics and Telecommunications, Peter the Great St. Petersburg Polytechnic University, 195251 St. Petersburg, Russia; mvin@spbstu.ru (M.Y.V.); vshalygin@spbstu.ru (V.A.S.); firsov.da@spbstu.ru (D.A.F.); 3 Department of Physics, Atmospheric Science & Geosciences, Jackson State University, Jackson, MS 39217, USA; lyudvig1977@gmail.com

**Keywords:** lens-shaped quantum dot, interband absorption, photoluminescence, second harmonic generation

## Abstract

The theoretical investigation of interband and intraband transitions in an asymmetric biconvex lens-shaped quantum dot are considered in the presence of an external magnetic field. The selection rules for intraband transitions are obtained. The behaviors of linear and nonlinear absorption and photoluminescence spectra are observed for different temperatures and magnetic field strengths. The second and third harmonic generation coefficients as a function of the photon energy are examined both in the absence and presence of an external magnetic field.

## 1. Introduction

Nanoparticles or quantum dots attract the attention of researchers due to their wide range of possible applications. One of these applications is the development of single photon emitters that can be used for quantum cryptography, optical quantum computing, and the development of highly secure communication networks [1]. Embedding the quantum dots into multilayer metamaterials and multilayer grating nanostructures can significantly increase the efficiency of such emitters due to the Purcell effect [2].

Possible optical transitions play an important role in the formation of the optical characteristics of nanoparticles. Thus, it was shown in [3] that interband optical transitions of charge carriers dominate the absorption and emission spectra in copper and gold nanocubes, while the plasmonic enhancement in these objects is less important to the emission. Along with this, enhanced intraband electron transitions can also be observed in plasmonic nanostructures. For example, in small gold nanocubes, intraband electron transitions make a significant contribution to photoluminescence, while interband transitions are the main contribution to the emission of large gold nanocubes [4]. In this regard, the analysis of optical effects associated with interband and intraband transitions of electrons in quantum dots of various types is an urgent problem.

Recent papers [5,6,7] reported the realization of GeSi quantum dots (QDs) with a strongly oblate lens-shaped geometry. In particular, the morphology of the structures was determined, and the specific optical characteristics of such QDs were revealed. An experimental confirmation of the implementation of Kohn theorem for the case of a heavy hole gas in strongly oblate lens-shaped QD was also found [6]. It is noteworthy that the specific geometry of QDs makes it possible to apply the adiabatic approximation for describing single-particle and few-particle states in such structures.

It is clear that for a description of the optical characteristics it is necessary to have detailed information about the band structure of studied QD. Therefore, the problem of constructing a realistic mathematical model of the investigated system becomes fundamentally important, which will affect the specific form of the one-particle or many-particle Hamiltonian. Since the geometry of the system is complicated and the separation of variables in the Schrödinger equation does not take place even in the case of a scalar effective mass, it is necessary to use various approximate methods to describe optical properties of strongly oblate lens-shaped QD. Many works are devoted to this problem in which both the spectral and optical characteristics of the QDs under study are discussed [8,9,10,11,12,13,14,15,16,17,18,19].

In [8], the authors considered optical transitions in a lens-shaped QD in the presence of hydrogenic impurity. The effect of the size of QD on the absorption coefficient has been investigated in the framework of density matrix formulation. Electronic and optical properties have been studied under external magnetic field in [10]. Authors have calculated the energy levels and wave functions using the finite element method (FEM) for different magnetic field values. Moreover, authors have studied the effect of the magnetic field on the second harmonic generation (SHG) and third-harmonic generation (THG) in the lens-shaped quantum dot. According to the obtained results, they found that the presence of the magnetic field affects the symmetry of the system. The influence of impurity on the binding energy and optical properties of lens-shaped quantum dots have been studied in [14]. The authors used FEM and the Arnoldi algorithm to calculate the absorption coefficient in the presence of impurity. The results showed that the binding energy decreased with QD size increase. Moreover, it has been shown that the absorption coefficient decreased, and the absorption peaks shifted toward the higher energies in the presence of the impurity. In another paper [15], the effects of QD sizes, pressure, and temperatures on transitions lifetime, linear, and nonlinear absorption coefficients in terahertz range were theoretically investigated. The authors considered two laterally coupled InAs/GaAs lens-shaped QDs connected to a wetting layer. The structure was analyzed by using the finite difference method (FDM) in the framework of effective mass approximation. The obtained results showed that the total absorption coefficient achieved a maximum value in the terahertz range, and the resonant peaks shifted toward the lower energies by increasing the pressure or decreasing the temperature. In addition to the influence of the magnetic field on the optical properties of a QD, the following factors can also affect the matrix in which the dot is embedded, electric fields, pressure, etc., [20,21,22,23]. In particular, in [23], the effect of pressure on interband and intraband transition of QDs was considered. The results showed that the interband and intraband transitions follow blue-shift and red-shift under pressure, respectively. The magnitude of the shift was, nevertheless, weaker in the intraband transitions case than for the interband one.

One of the powerful mechanisms for studying the QD band structure is a comprehensive analysis of the linear and nonlinear absorption spectra of the investigated structures [24,25,26,27,28,29,30,31,32]. As indicated above, using the adiabatic approximation, one-particle states can be successfully described, in particular, in the presence of external fields [6,33,34]. The analytical form of the energy spectrum and wave functions for electrons and holes makes it possible to give a complete and comprehensive description of the optical properties of strongly oblate lens-shaped QD. This, in turn, makes it possible to draw a number of conclusions regarding the specificity of the band structure of such structures.

In this article, in the framework of adiabatic approximation the linear and nonlinear optical absorption and photoluminescence related to interband and intraband optical electron transitions in strongly oblate lens-shaped InAs QD in the presence of external magnetic field are considered. Note an important feature of the considered model. In the axial direction size, quantization is much higher than in radial one. It is clear that when in the presence of an axial magnetic field, we must take into account such fields at which the axial subsystem will be fast, since, in the case of strong magnetic fields, the role of magnetic quantization can become more significant in comparison with size quantization in the axial direction. As a result, the slow and fast subsystems are reversed. We do not cover such magnetic fields in this article. However, we note that in the case of such high fields, the energy spectrum will be characterized by a subband structure where a family of axial levels will be associated with each Landau level.

## 2. Theoretical Model

**Figure 1:**
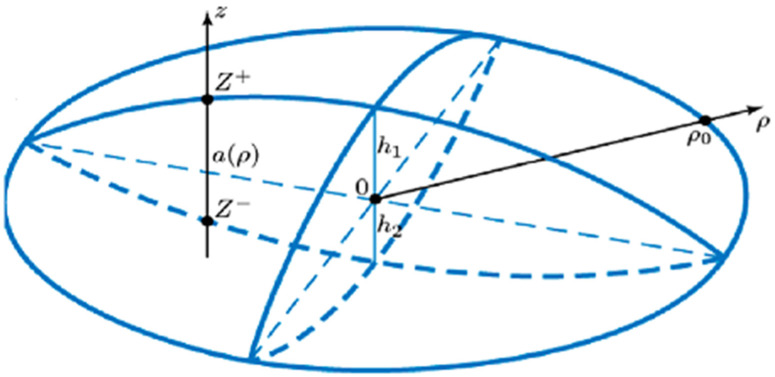
Schematic of the asymmetric biconvex lens-shaped QD.

Let us consider the electron states in an asymmetric biconvex lens-shaped QD (Figure 1) in the presence of an external axial magnetic field. The confining potential has been chosen in following form

(1) $$U_{conf}\left(\vec{r}\right)=\left\{ \begin{array}{l} 0,\;-\sqrt{R_{2}^{2}-\rho^{2}}+R_{2}-h_{2}<z<\sqrt{R_{1}^{2}-\rho^{2}}-R_{1}+h_{1}\\ \infty ,\;other\;cases \end{array}\right.$$

where $h_{1,2}$ are semi-axes for each convex part of asymmetric biconvex lens-shaped QD, $R_{1,2}$ are the curvature radii of two spheres intersection.

Along the axial direction, the particle is localized in the one-dimensional quantum well with following boundaries [5,34]

(2) $$\left\{ \begin{array}{l} z^{+}=\sqrt{R_{1}^{2}-\rho^{2}}-R_{1}+h_{1}\\ z^{-}=-\sqrt{R_{2}^{2}-\rho^{2}}+R_{2}-h_{2} \end{array}\right.$$

The Hamiltonian of the system has the form:

(3) $$\hat{H}=\frac{1}{2m^{*}}{\left(\hat{\vec{p}}+\frac{e}{c}\vec{A}\right)}^{2}+U_{conf}\left(\vec{r}\right),$$

where $m^{*}$ is the effective mass of the particle, $\hat{\vec{p}}$ is the particle momentum operator, e is the electron charge magnitude, c is the speed of light in vacuum, and $\vec{A}$ is the vector potential of the magnetic field.

The calibration of the vector potential in cylindrical coordinates was chosen as $\vec{A}=\left\{0,0,H\rho /2\right\}$, where $\vec{H}$ is the magnetic field strength.

## 3. Energy Spectra and Wave Functions

For the Schrödinger equation of problem (3) with confining potential (1) in cylindrical coordinates we have

(4) $$-\frac{\hbar^{2}}{2m^{*}}\left[\frac{\partial^{2}}{\partial \rho^{2}}+\frac{1}{\rho }\frac{\partial }{\partial \rho }+\frac{1}{\rho^{2}}\frac{\partial^{2}}{\partial \varphi^{2}}+\frac{\partial^{2}}{\partial z^{2}}\right]\psi -\frac{i\hbar \omega_{H}}{2}\frac{\partial \psi }{\partial \varphi }+\frac{m^{*}\omega_{H}^{2}\rho^{2}}{8}\psi +U_{conf}\psi =E\psi ,$$

where $\omega_{H}=\frac{eH}{m^{*}c}$ is the cyclotron frequency.

In adiabatic approximation, the total wave function of the system is searched in the following form [34]:

(5) $$\psi \left(r,\varphi ,z\right)=\frac{1}{\sqrt{2\pi }}e^{im\varphi }f\left(\rho \right)\chi \left(z;\rho \right),$$

where m=0, ±1, ±2, … is the magnetic quantum number. According to (2) and (4), for χ(z;ρ) we have

(6) $$\chi \left(z,\rho \right)=\sqrt{\frac{2}{a\left(\rho \right)}}\sin\left(\frac{\pi n_{z}}{a\left(\rho \right)}\left(z+\sqrt{R_{2}^{2}-\rho^{2}}+h_{2}-R_{2}\right)\right)$$

where $a\left(\rho \right)=\sqrt{R_{2}^{2}-\rho^{2}}+\sqrt{R_{1}^{2}-\rho^{2}}+\left(h_{1}+h_{2}\right)-\left(R_{1}+R_{2}\right)$ is one-dimensional quantum well length, $n_{z}=1,\;2,\;3,\;\ldots$ is axial quantum number. For energy spectrum we have

(7) $$E_{n_{z}}=\frac{\pi^{2}\hbar^{2}}{2m^{*}a^{2}\left(\rho \right)}n_{z}^{2}$$

Axial energy plays the role of effective potential for radial one. Placing (7) in the Taylor series around ρ=0 for $U_{eff}$, we obtain

(8) $$U_{eff}\left(\rho \right)=\frac{\pi^{2}\hbar^{2}n_{z}^{2}}{2m^{*}{\left(h_{1}+h_{2}\right)}^{2}}+\frac{m^{*}\omega_{0}^{2}\rho^{2}}{2}$$

where $\omega_{0}^{2}=\frac{R_{1}+R_{2}}{{\left(m^{*}\right)}^{2}{\left(h_{1}+h_{2}\right)}^{3}R_{1}R_{2}}\pi^{2}\hbar^{2}n_{z}^{2}$. For the radial Schrödinger equation, we can write

(9) $$-\frac{\hbar^{2}}{2m^{*}}\left[\frac{\partial^{2}}{\partial \rho^{2}}+\frac{1}{\rho }\frac{\partial }{\partial \rho }-\frac{m^{2}}{\rho^{2}}\right]f\left(\rho \right)+\frac{m^{*}\Omega^{2}}{2}\rho^{2}f\left(\rho \right)=\varepsilon_{n_{\rho },m}f\left(\rho \right),$$

where $\varepsilon_{n_{\rho },m}=E_{n_{\rho },m}-\frac{\pi^{2}\hbar^{2}n_{z}^{2}}{2m^{*}{\left(h_{1}+h_{2}\right)}^{2}}-\frac{m\hbar \omega_{H}}{2}$, $\Omega^{\;2}=\frac{\omega_{H}^{2}}{4}+\omega_{0}^{2}$. The solution of (9) is well known, and for the wavefunction, we have

(10) $$f\left(\rho \right)=C\rho^{\left|m\right|}e^{-\frac{\lambda \rho^{2}}{2}}{}_{1}F_{1}\left(-n_{\rho },\left|m\right|+1;\lambda \rho^{2}\right)$$

where $\lambda =\frac{m^{*}\Omega }{\hbar }$, $n_{\rho }=0,\;1,\;2,\;\ldots$ is the radial quantum number, C is the wavefunction normalization constant, and ${}_{1}F_{1}\left(a,b;c\right)$ is the confluent hypergeometric function. For the energy spectrum, we can write

(11) $$E_{n_{\rho },m}=\hbar \Omega \left(2n_{\rho }+\left|m\right|+1\right)+\frac{\pi^{2}\hbar^{2}n_{z}^{2}}{2m^{*}{\left(h_{1}+h_{2}\right)}^{2}}+\frac{m\hbar \omega_{H}}{2}$$

Note that all results were obtained for relatively weak magnetic fields, when the size quantization in the axial direction is much higher than the magnetic field intensity.

## 4. Results

As was mentioned above, the case of the asymmetric biconvex lens-shaped QD made from *InAs* was considered. The material parameters used in the calculations were the following:$m_{e}^{*}=0.023m_{0}$ is the electron effective mass, $m_{lh}^{*}=0.026m_{0}$, $m_{hh}^{*}=0.41m_{0}$ are the effective masses of the light and heavy holes, respectively, where $m_{0}$ is the free electron mass, $\varepsilon_{r}=15.5$ [35,36]. Using the Varshni relation, the temperature dependence of the bandgap in *InAs* can be described as $E_{g}\left(T\right)=E_{g}\left(0\right)+\frac{\alpha T^{2}}{\left(T+\beta \right)}$, where $E_{g}\left(0\right)=415\;meV$ and α=0.276 meV/K, β=83 K [37].

Let us consider the direct interband absorption in the strong size quantization regime, when the Coulomb interaction between electron and hole can be neglected. Then, the absorption coefficient is given by [38]

(12) $$\alpha \left(\hbar \omega \right)=\alpha_{0}\sum_{\nu_{e},\nu_{h}}{\left|\int \Psi_{e}\left(\vec{r}\right)\Psi_{h}\left(\vec{r}\right)dV\right|}^{2}\delta \left(\hbar \omega -E_{g}\left(T\right)-E_{\nu_{e}}^{e}-E_{\nu_{h}}^{h}\right)$$

where $\alpha_{0}$ is the quantity proportional to the square of the modulus of the matrix element of the dipole moment taken over the Bloch functions, $\Psi_{e}\left(\vec{r}\right),\;\Psi_{h}\left(\vec{r}\right)$ are electron and hole wave functions, $E_{g}\left(T\right)$ is temperature dependence of the energy gap, $E_{\nu_{e}}^{e}\left(E_{\nu_{h}}^{h}\right)$ is electron (hole) energy, $\nu_{e}\left(\nu_{h}\right)$ is set of quantum numbers for electron (hole), and ℏω is incident photon energy. The broadening was taken into account within the framework of the Lorentz model. For this purpose, the delta function in (12) was replaced by the Lorentz contour with the broadening parameter Γ. For the dependence of the broadening linewidth Γ on the temperature, the following equation was constructed $\Gamma \left(T\right)=\Gamma \left(0\right)+A\cdot T+B\cdot T^{2}$, where ℏΓ(0)=1.459meV, A=0.00138 meV/K, $B=0.00005\;meV/K^{2}$ [39].

**Figure 2:**
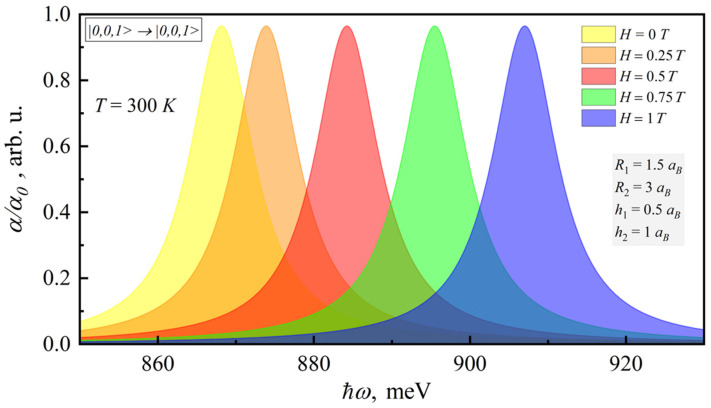
Dependence of the room-temperature interband absorption coefficient on the photonenergy for different magnetic fields.

Figure 2 shows the dependence of the interband absorption coefficient for $|m,n_{\rho },n_{z}>\to |m^{\prime },{n^{\prime }}_{\rho },{n^{\prime }}_{z}>$ transition on the photon energy. Note that the following selection rules were obtained

(13) $$\left\{ \begin{array}{l} m\to -m^{\prime }\\ n_{z}\to {n^{\prime }}_{z}\\ n_{\rho }\to {n^{\prime }}_{\rho } \end{array}\right.$$

In this case, the transitions non-diagonal in $n_{z}$ or $n_{\rho }$ have no probability; therefore, they are not shown in the figure. It can be seen from Figure 2 that the account of the magnetic field brings the blue shift of the interband absorption peak.

**Figure 3:**
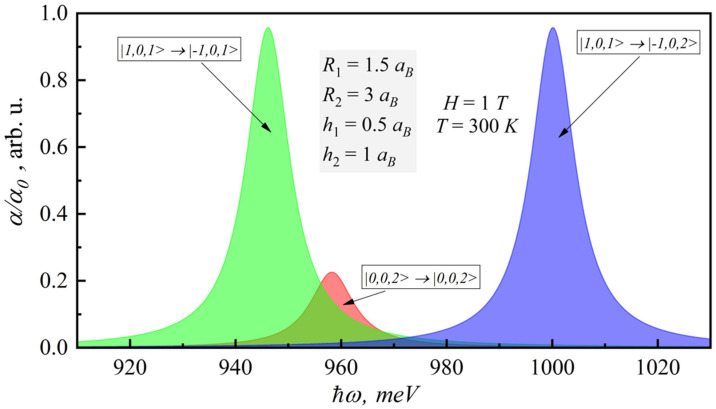
Dependence of the room-temperature interband absorption coefficient on photonenergy for different interband transitions.

Figure 3 shows the dependence of the absorption coefficient for different interband transitions on the photon energy. In this case, the transitions non-diagonal in $n_{z}$ or $n_{\rho }$ have no probability; therefore, they are not shown in Figure 3.

**Figure 4:**
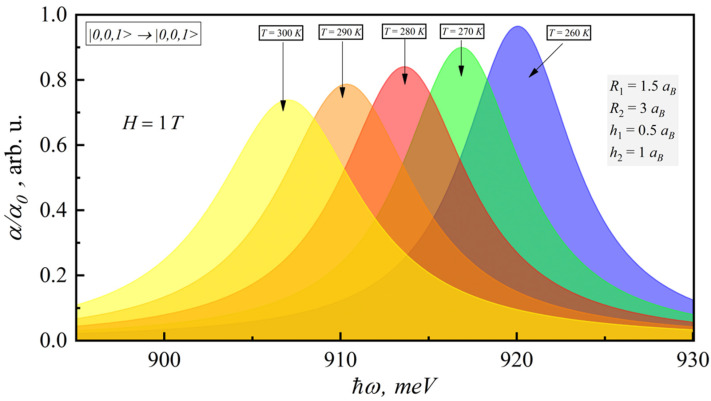
Dependence of room-temperature interband absorption coefficient on photonenergy for different temperatures.

Figure 4 presents the dependence of the interband absorption coefficient for |0,0,1〉→|0,0,1〉 transition on the photon energy for different temperatures (260–300 K). As can be seen from the figure, interband absorption also increases with temperature for constant magnetic field. The account of temperature brings the red shift of interband absorption.

The photoluminescence (PL) coefficient is calculated using the relation [40,41]

(14) $$R\left(\hbar \omega \right)=R_{0}\cdot \hbar \omega \cdot \alpha \left(\hbar \omega \right)\cdot \frac{f_{c}\left(1-f_{v}\right)}{f_{c}-f_{v}}$$

where $R_{0}$ is the quantity proportional to the square of the modulus of the matrix element of the dipole moment, taken over the Bloch functions, k is the Boltzmann constant, $f_{c}$ and $\left(1-f_{v}\right)$ are the probabilities that the state of the conduction band is filled and the state of the valence band is empty, respectively.

**Figure 5:**
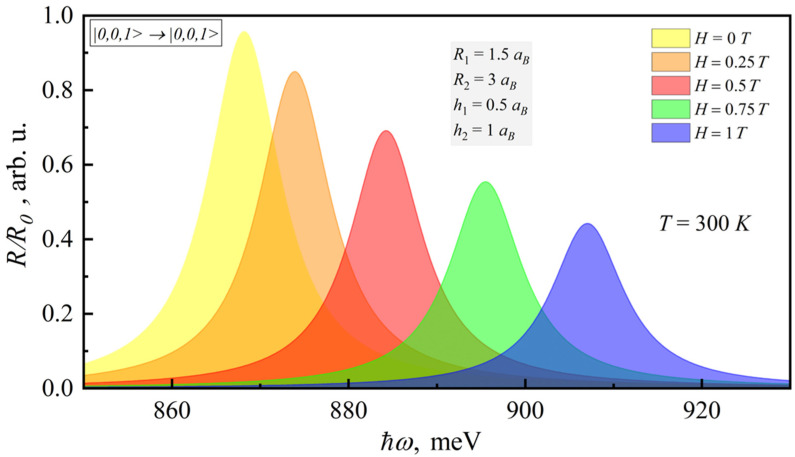
Dependence of the room-temperature PL coefficient on photonenergy for different magnetic fields.

Figure 5 shows the dependence of the PL coefficient on the photon energy for different the magnetic field strengths. The behavior of the PL coefficient with respect to temperature and magnetic field is similar to the behavior of the absorption coefficient.

**Figure 6:**
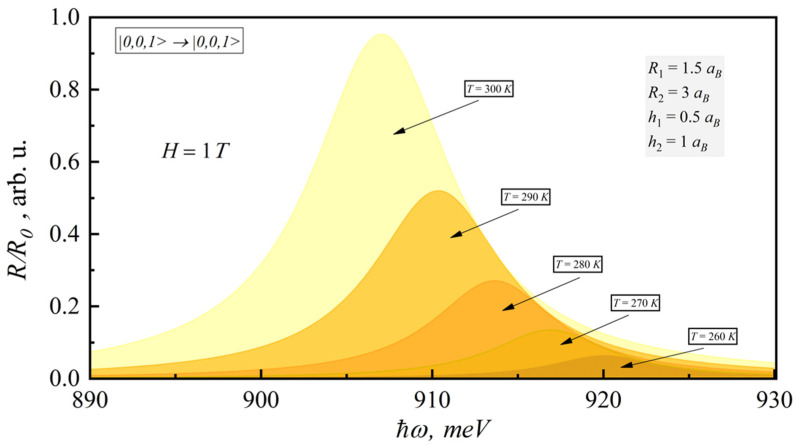
Dependence of the room-temperature PL coefficient on photon energy for different temperatures.

Figure 6 demonstrates the dependence of the PL coefficient on the photon energy for different temperatures, close to room temperature. The PL coefficient significantly increases upon increasing the temperature in a rather narrow range from 260 to 300 K.

Now let us consider the direct intraband light absorption. Analytical expressions for the linear and nonlinear optical absorption coefficients are obtained, using the compact density matrix approach and iterative method [42,43]. Thus, the linear and third-order nonlinear optical absorption coefficients of a QD can be written as:

(15) $$\alpha^{\left(1\right)}\left(\omega \right)=\omega \cdot \sqrt{\frac{\mu }{\varepsilon_{r}}}\frac{\sigma \cdot \hbar \Gamma }{{\left(E_{fi}-\hbar \omega \right)}^{2}+{\left(\hbar \Gamma \right)}^{2}}{\left|M_{fi}\right|}^{2},$$

(16) $$\begin{array}{l} \alpha^{\left(3\right)}\left(\omega ,I\right)=-\omega \sqrt{\frac{\mu }{\varepsilon_{r}}}\left(\frac{I}{\varepsilon_{0}n_{r}c}\right)\frac{{\left|M_{fi}\right|}^{4}2\sigma \cdot \hbar \Gamma }{{\left[{\left(E_{fi}-\hbar \omega \right)}^{2}+{\left(\hbar \Gamma \right)}^{2}\right]}^{2}}\times \\ \times \left\{1-\frac{{\left|M_{ii}-M_{ff}\right|}^{2}}{4{\left|M_{fi}\right|}^{2}}\left(\frac{{\left(E_{fi}-\hbar \omega \right)}^{2}-{\left(\hbar \Gamma_{fi}\right)}^{2}+2E_{fi}\left(E_{fi}-\hbar \omega \right)}{{\left(E_{fi}\right)}^{2}+{\left(\hbar \Gamma \right)}^{2}}\right)\right\}, \end{array}$$

where μ is the permeability of the system, σ is the electron density in a QD, $E_{fi}=E_{f}-E_{i}$ is energy difference between the final and initial states (f and i, respectively), Γ=1/τ is the relaxation rate for states f and i (corresponds to the full width at half maximum), I is the incident optical intensity, $M_{fi}=\langle \Psi_{f}|ez|\Psi_{i}\rangle$ is the matrix element of dipole moment, and $n_{r}$ is the refractive index.

**Figure 7:**
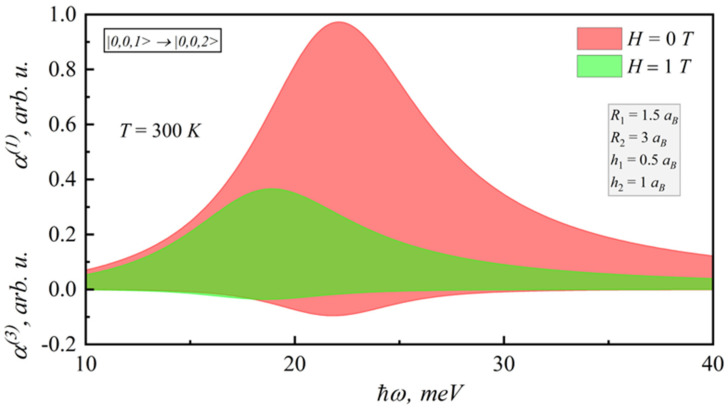
Spectral dependences of the linear and nonlinear intraband absorption coefficients on photon energy for different magnetic fields.

**Figure 8:**
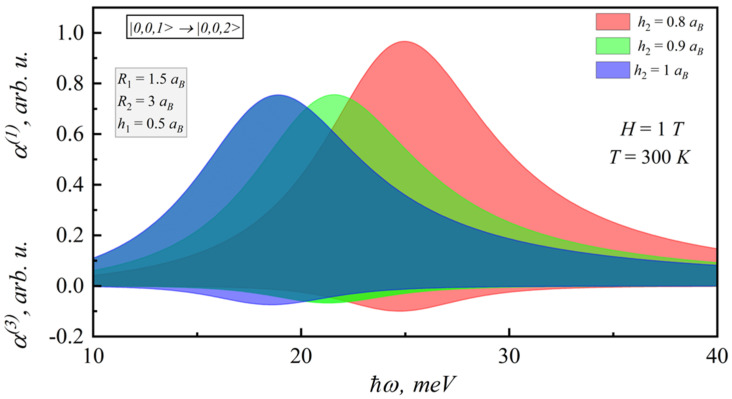
Spectral dependences of the linear and nonlinear intraband absorption coefficients on photon energy for different lens-shaped QD heights.

For the spectral dependences of the linear and nonlinear coefficients for different magnetic fields and lens-shaped QD heights on the photon energy, when incident optical intensity is I = 1 kW/cm^2^ and electron density in QD σ = 3·10^16^ cm^−^^3^ [44,45], the nonlinear absorption has the opposite sign to the linear one. Calculations were for 300 K. First of all, it should be mentioned that the nonlinear absorption value decreases, but the nonlinear practically does not change (see Figure 7 and Figure 8).

The expression for the SHG susceptibility in a three-level system is given by [42,43]:

(17) $$\chi^{\left(2\right)}\left(2\omega \right)=\frac{e^{3}\sigma }{\varepsilon_{0}}\frac{M_{in_{1}}M_{n_{1}f}M_{fi}}{\left(2\hbar \omega -E_{fi}-i\hbar \Gamma_{fi}\right)\left(\hbar \omega -E_{n_{1}i}-i\hbar \Gamma_{n_{1}i}\right)}$$

The THG susceptibility in a four-level system is [42,43]:

(18) $$\chi^{\left(3\right)}\left(3\omega \right)=\frac{e^{4}\sigma }{\varepsilon_{0}}\frac{M_{in_{1}}M_{n_{1}n_{2}}M_{n_{2}f}M_{fi}}{\left(3\hbar \omega -E_{fi}-i\hbar \Gamma_{fi}\right)\left(2\hbar \omega -E_{n_{2}i}-i\hbar \Gamma_{n_{2}i}\right)\left(\hbar \omega -E_{n_{1}i}-i\hbar \Gamma_{n_{1}i}\right)}$$

where $n_{1}$ and $n_{2}$ are quantum numbers of intermediate levels.

**Figure 9:**
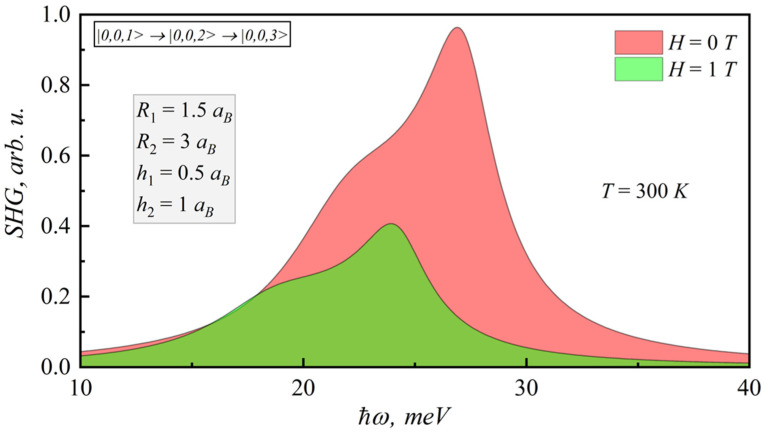
Dependence of SHG coefficient on photonenergy for different magnetic fields.

**Figure 10:**
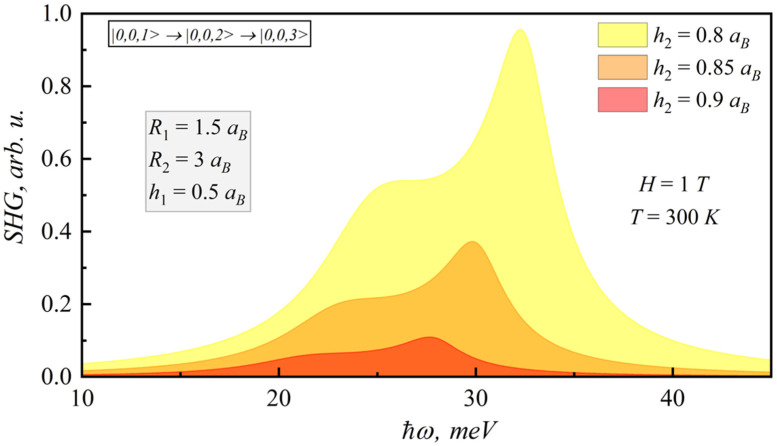
Dependence of SHG coefficient on photonenergy for different lens-shaped QD heights.

**Figure 11:**
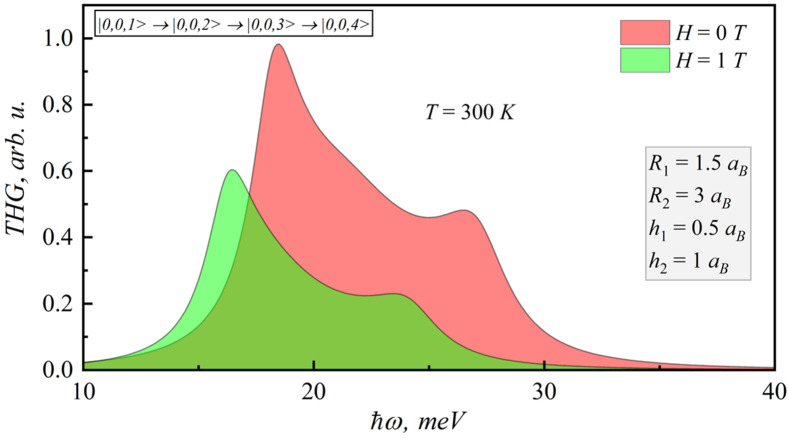
Dependence of THG coefficient on photonenergy for different magnetic fields.

**Figure 12:**
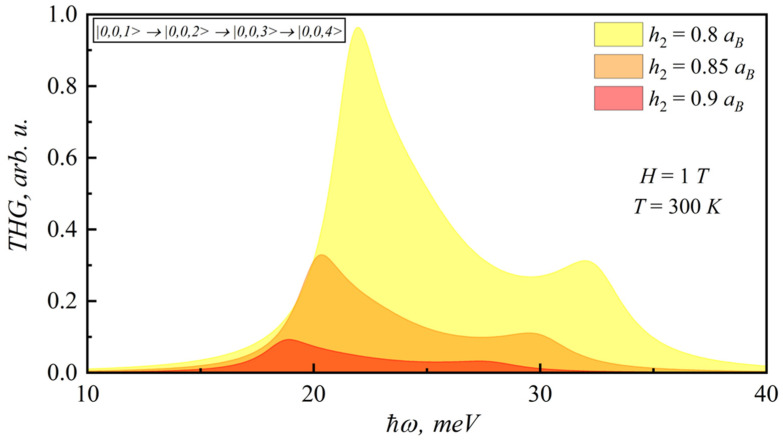
Dependence of THG coefficient on photon energy for different lens-shaped QD heights.

In the Figure 9, Figure 10, Figure 11 and Figure 12, the SHG and THG curves are presented for different magnetic field values of photon energy. Calculations were performed for 300 K. It should be noted that an increase in the magnetic field strength leads to a decrease in both SHG and THG peaks, since the nonlinearity of the structure decreases.

## 5. Conclusions

In this paper, the interband and intraband optical transitions in an asymmetric biconvex lens-shaped QD made in InAs in the presence of an external magnetic field were investigated. An advantage of our approach is the possibility of using an analytical method for describing electron and hole states in a strongly oblated quantum lens. This approach made it possible to obtain explicitly the selection rules for interband transitions. A study of the interband and intraband absorption coefficients showed a broadening of the absorption peaks with increasing temperature and a decrease in the peaks amplitudes with magnetic field strength increasing. The SHG and THG nonlinear parameters of the system decreased with an increase in the magnetic field and/or temperature. Our results highlighted the significant difference between linear and nonlinear absorptions (calculations showed that the contribution of the linear absorption significantly exceeded the contribution of the nonlinear one. With a magnetic field increase the interlevel distances also increased, as a result of which, the overlap of the wave functions weakened. Therefore, the contribution of the corresponding matrix elements decreased, while this was more significant for nonlinear absorption). Moreover, magnetic field shifted the absorption peaks to higher light frequencies in the case of interband transitions and to low frequencies in the case of intraband transitions. In the case of relatively low temperatures, SHG and THG can be clearly observed. The results obtained above suggest that the temperature and magnetic field significantly affect the optical properties of the considered nanostructure.

## Data Availability

The data that support the findings of this study are available from the corresponding author upon reasonable request.
